# HDAC 1 and 6 modulate cell invasion and migration in clear cell renal cell carcinoma

**DOI:** 10.1186/s12885-016-2604-7

**Published:** 2016-08-09

**Authors:** Swathi Ramakrishnan, ShengYu Ku, Eric Ciamporcero, Kiersten Marie Miles, Kris Attwood, Sreenivasulu Chintala, Li Shen, Leigh Ellis, Paula Sotomayor, Wendy Swetzig, Ray Huang, Dylan Conroy, Ashley Orillion, Gokul Das, Roberto Pili

**Affiliations:** 1 Department of Cancer Pathology and Prevention, Roswell Park Cancer Institute, Buffalo, NY USA; 2 Genitourinary Program, Roswell Park Cancer Institute, Buffalo, NY USA; 3 Department of Pharmacology and Therapeutics, Roswell Park Cancer Institute, Buffalo, NY USA; 4 Department of Medicine and Experimental Oncology, University of Turin, Turin, Italy; 5 Department of Biostatistics & Bioinformatics, Roswell Park Cancer Institute, Buffalo, NY USA; 6 Center for Integrative Medicine and Innovative Science-Universidad Andres Bello, Santiago, Chile; 7 Department of Cancer Genetics, Roswell Park Cancer Institute, Buffalo, NY USA; 8 Genitourinary Program, Indiana University- Simon Cancer Center, Indianapolis, IN USA

## Abstract

**Background:**

Class I histone deacetylases (HDACs) have been reported to be overexpressed in clear cell renal cell carcinoma (ccRCC), whereas the expression of class II HDACs is unknown.

**Methods:**

Four isogenic cell lines C2/C2VHL and 786-O/786-OVHL with differential VHL expression are used in our studies. Cobalt chloride is used to mimic hypoxia *in vitro*. HIF-2α knockdowns in C2 and 786-O cells is used to evaluate the effect on HDAC 1 expression and activity. Invasion and migration assays are used to investigate the role of HDAC 1 and HDAC 6 expression in ccRCC cells. Comparisons are made between experimental groups using the paired *T*-test, the two-sample Student’s *T*-test or one-way ANOVA, as appropriate. ccRCC and the TCGA dataset are used to observe the clinical correlation between HDAC 1 and HDAC 6 overexpression and overall and progression free survival.

**Results:**

Our analysis of tumor and matched non-tumor tissues from radical nephrectomies showed overexpression of class I and II HDACs (HDAC6 only in a subset of patients). In vitro, both HDAC1 and HDAC6 over-expression increased cell invasion and motility, respectively, in ccRCC cells. HDAC1 regulated invasiveness by increasing matrix metalloproteinase (MMP) expression. Furthermore, hypoxia stimulation in VHL-reconstituted cell lines increased HIF isoforms and HDAC1 expression. Presence of hypoxia response elements in the HDAC1 promoter along with chromatin immunoprecipitation data suggests that HIF-2α is a transcriptional regulator of HDAC1 gene. Conversely, HDAC6 and estrogen receptor alpha (ERα) were co-localized in cytoplasm of ccRCC cells and HDAC6 enhanced cell motility by decreasing acetylated α-tubulin expression, and this biological effect was attenuated by either biochemical or pharmacological inhibition. Finally, analysis of human ccRCC specimens revealed positive correlation between HIF isoforms and HDAC. HDAC1 mRNA upregulation was associated with worse overall survival in the TCGA dataset.

**Conclusions:**

Taking together, these results suggest that HDAC1 and HDAC6 may play a role in ccRCC biology and could represent rational therapeutic targets.

**Electronic supplementary material:**

The online version of this article (doi:10.1186/s12885-016-2604-7) contains supplementary material, which is available to authorized users.

## Background

Inactivation of the tumor suppressor gene von Hippel Lindau, VHL, is a common alteration in sporadic clear cell renal cell carcinomas (ccRCCs) [1]. VHL protein is responsible for the proteasomal degradation of hypoxia inducible factors (HIF) by binding to the oxygen dependent domain on HIF, thus inhibiting downstream target genes involved in angiogenesis, glycolysis and cell cycle [2–4].

Histone deacetylases (HDACs), enzymes that regulate chromatin status and gene expression, are subdivided into four classes (I, II, III and IV), based on their structure [5]. In ccRCC, class I HDACs (i.e. HDAC 1 and HDAC 2) have been reported to be overexpressed, as compared to adjacent non-tumor tissues [6]. Our lab has shown that class II HDACs, HDAC 4 and HDAC 6, stabilize HIF-1α in renal and prostate tumor cells [7, 8]. However, studies related to the regulation of HDAC expression and the role of HDACs in ccRCC tumor biology remain limited.

Class I HDACs, specifically HDAC 1, is upregulated at both the mRNA and protein level under hypoxic conditions, which corresponds to increased HDAC activity that can be blocked by the HDAC inhibitor (HDACi) trichostatin A (TSA) [9]. Class I HDACs further regulate HIF-1α stability, and TSA abrogates this effect in HeLa cells [10]. The pan HDACi panobinostat downregulates HIF-1α protein in HUVECs as well as in prostate cancer cell lines under normoxic and hypoxic conditions [11]. The class II HDAC 6 increases invasiveness and motility in kidney epithelial cells through deacetylation of α-tubulin, which is counteracted by a specific HDAC 6 inhibitor (tubacin) and TSA [12]. HDAC 6 translocation to the plasma membrane is associated with membrane estrogen receptor alpha (ERα), and deacetylation of α-tubulin increases motility of breast tumor cells *in vitro* [13]. HDAC 6 upregulation in MCF7 cells changed the morphological features as well as the migration capacity of these cells [14]. In addition, estrogen receptor (ER)-positive tumors with concomitant HDAC 6 overexpression showed significant increase in overall and cancer specific survival after tamoxifen treatment [14]. Early evidence for the expression of ERα in kidney tumors has been demonstrated in an estradiol-induced hamster kidney tumor model that showed the presence of ERα in epithelial tumor cells and stromal cells in both female and male hamsters [15].

There are several studies involving the combination of HDAC inhibitors and ERα antagonists in breast cancer. In ER-positive tumors, panobinostat increases cell death in synergy with hydroxytamoxifen [16], whereas valproic acid in combination with tamoxifen augmented the inhibition of cell proliferation and apoptosis [17]. TSA also enhanced the effectiveness of hormonal therapy in ER-negative breast tumors through ERβ activity [18]. Additionally, RCC cells when treated with estrogen showed decreased proliferation, migration and invasion of cells, primarily through ERβ effects [19].

In this study, we investigated the role of class I and II HDACs in ccRCC tumor biology by utilizing *in vitro* models and human samples.

## Methods

### Cell lines, treatments and antibodies

Renal cell lines C2, C2VHL and 786–0 were kindly provided by Drs. Jennifer Isaacs and Len Neckers (National Cancer Center). Cells were cultured in DMEM media supplemented with 10 % FBS at 37 °C and 5 % CO_2_ concentration. 5x10^5^ cells in duplicate 12-well plates were serum-starved for 24 h followed by treatment with media/10 % FBS with or without the hypoxia. Cobalt chloride (100 μM) (Sigma Aldrich, Cat.no. 232696) addition for 24 h was used as hypoxia mimic in these studies. At the designated time point, cells were harvested in RIPA buffer (Sigma Aldrich, Cat. no. R0278) with protease and phosphatase inhibitors (Roche) for western blot. For short term effects on the levels of acetylated alpha tubulin, 3000 cells were plated on coverslips overnight, followed by treatment with hydroxytamoxifen (Sigma Aldrich, Cat. no. T176) and/or panobinostat (Novartis) for 4 h. Antibodies against HIF-1α (Cayman chemical, Cat.no. 10006421), HIF-2α (Abcam, Cat.no. ab199), HDAC 1 (Cell signaling, Cat.no. 5356), acetylated H3 (Millipore, Cat.no. 06–599), HDAC 6 (Santacruz Cat. no. sc-11420), ER-alpha (Santacruz, Cat. no. sc-543), acetylated α-tubulin (Life technologies, Cat. no. 32–2700), total histone H3 (Cell signaling, Cat.no. 9715), GAPDH (Cell signaling, Cat. No. 2118), and HRP-conjugated rabbit (BioRad, Cat.no. 170–6515) and mouse (Dako, Cat.no. P0260) secondary antibodies were used at the recommended dilutions.

### Western blot analysis and flow cytometry

Cells were harvested using RIPA buffer for Western blot, and 40 μg of total protein were run on 12 % gels followed by wet transfer at 25 V overnight at room temperature. The blots were then blocked with 10 % milk, followed by incubation with primary antibody and HRP-conjugated secondary antibody. Protein bands were detected with ECL (Perkin Elmer, Cat.no. NEL105001EA). 8x10^5^ cells were plated for flow cytometry, treated and harvested for fixation and permeabilization (BD Pharmingen, Cat. no. 560409). Cells were blocked with blocking serum, incubated with HDAC 1 antibody, washed, incubated with secondary FITC-conjugated anti-mouse antibody (BD bioscience, Cat.no. 554001) and finally stained with propidium iodide for cell cycle analysis. Cells were run on a LSR Fortessa, and results were analyzed using FCS Express software.

### Transfections

The wt-VHL plasmid was kindly provided Dr. Michael Ohh (University of Toronto) and transfected into 786–0 cells with Lipofectamine 2000 (Life technologies, Cat.no. 11668–019) and OptiMEM media (Life Technologies, Cat. no. 31985070). The following day, cells were incubated with media containing neomycin and selected for two weeks for stable transfection. The HDAC 6 plasmid (kindly provided by Dr. Tso Pang Yao at Duke University) and the HDAC 1 shRNA were transfected and packaged in retroviral cells at the RPCI genomics core facility. Retroviral supernatants were added to C2 and 786–0 cells, spun for 45 min at 1800 rpm and incubated for 4 h at 37 °C. Regular medium was then added to the cells, and puromycin (for HDAC 1 knockdown selection) or neomycin (for HDAC 6 selection) was added for selection the next day. Cells that were infected were selected for a period of two weeks. HDAC 1 and HDAC 6 knockdown was observed by Western blot and immunofluorescent analysis, respectively. For HIF-2α knockdown, shRNA against HIF-2α was purchased from Addgene (Plasmid 22131) and transfected using retroviral supernatants generated at the RPCI genomics core facility. The next day, cells were incubated with regular media and selected with neomycin for a period of two weeks. The cells were tested for HIF-2α knockdown efficiency by Western blot analysis. For ERα knockdown, siRNA against ERα was transfected using Lipofectamine 2000 in OptiMEM media. The cells were tested for ERα knockdown efficiency by Western blot, and acetylated α-tubulin levels were measured by immunofluorescence.

### Proliferation and Invasion assay

For proliferation assays, 8x10^3^ cells were plated in 24 well plates with regular media and harvested after 24, 48 and 72 h for measurement of proliferation by staining the wells with crystal violet. This was followed by dissolution of the stain in methanol for 2–3 h, and the plates were read at 590 nm. Proliferation at different time points was compared to 24 h for growth rate calculations. For invasion assays, 5x10^5^ cells were plated on top of Matrigel-coated chambers (BD bioscience, Cat.no. 354480) in regular medium with serum overnight. The medium on top was replaced with serum free media the next day, and media with serum was added to the bottom of the well as a chemoattractant. The non-invading cells at the top of the chamber were removed with cotton swabs (after 4 h for 786–0 cells and 24 h for C2 cells), and cells on the lower surface were stained with crystal violet (Sigma Aldrich, Cat. no. HT 90132) for 30 min. The inserts were washed thrice with distilled water, and the number of invading cells were counted by observation under the microscope. The HDAC 1 knockdown cells were compared to the parental cell lines for measuring invasion capability. In addition, a gelatin zymography assay was performed to analyze the matrix metalloprotease (MMP) activity in the cell lysate as well as in the supernatant. Briefly, 5x10^4^ cells were plated in a 24-well plate in regular DMEM with serum for 24 h. This was followed by media change to DMEM without serum (to analyze MMP activity in the cell supernatant) for 16 h. Cell supernatants and cell lysates (harvested by RIPA Buffer) were collected at the end of the experiment. The lysates and supernatants were then run on 7.5 % acrylamide gels with 1 % gelatin (substrate for MMP); followed by incubation with renaturing buffer for 30 min, developing buffer overnight at 37 °C, stained with commassie blue for 30 min, and finally destained with destaining solution until the bands on the gel were strong and clear.

### Immunofluorescence assay

After treatments, cells were fixed in 4 % formaldehyde, followed by permeabilization with Triton-X 100 (Sigma Aldrich; Cat. no. T8787) and blocking with 1 % bovine serum albumin for one hour. The cells were stained for acetylated α-tubulin, HDAC 6 and ERα and detected by secondary FITC or Alexa-fluor tagged secondary antibody. Zeiss AxioImager and the axiovision software were used to capture immunofluorescent images at 20X magnification. Immunofluorescent images were analyzed using the NIH software Image J. Integrated density of images was calculated using Image J and plotted as bar graphs for comparison of intensity of fluorescence in images.

### Motility and migration assay

5x10^5^ cells were plated in a 12-well plate overnight to develop a monolayer, followed by creating a horizontal scratch in the plate using a sterile pipette tip. The cells were then placed in fresh media and observed over a period of 24 h. A grid was developed for the 12-well plate to maintain the same field of observation for cell motility.

### Analysis of HDAC 1 promoter and non-promoter region by Chromatin immunoprecipitation assay

1x10^6^ cells were plated in 10 cm^2^ culture dishes overnight, and the ChIP protocol from Novus was followed. Briefly, cells were fixed with 1 % formaldehyde (Sigma Aldrich, Cat. no. 252549), and 125 mM glycine (Sigma Aldrich, Cat.no. G8898) was added to the media for quenching, followed by washing with PBS and harvesting of the cells with RIPA buffer. The samples were then sonicated for twelve 15 s pulses at a 50 % output with a 60 s rest on ice, centrifuged to remove debris, and then incubated with HIF-1α, HIF-2α or corresponding Ig antibodies overnight. Magnetic Protein G beads (Invitrogen, Cat.no. 10003D) were added to the samples overnight at 4 °C, washed and eluted with IP elution buffer and finally incubated with proteinase K overnight at 62 °C. cDNAs were isolated with Phenol/chloroform/isoamyl alcohol followed by qPCR of immunoprecipitated samples. Input control and percent input were calculated and compared with Ig controls. Primers used for qPCR analysis were as follows:

- HDAC 1 promoter region: Forward primer: 5’-GACCGACTGACGGTAGGGA-3’, Reverse primer: 5’-GGTGCTCACCGTCGTAGTAG-3’
- HDAC 1 non-promoter region: Forward primer: 5’-GAGTGTGCAGGTTCTGCTCT-3’, Reverse primer: 5’-CACACCCAGCCAGACTGAAT-3’
- VEGF promoter region: Forward primer: 5’-GATCTGTGTGTCCCTCTCCC-3’, Reverse primer: 5’-AAAGTGAGGTTACGTGCGGA-3’

### Immunohistochemistry and Immunofluorescence of TMA

A small cohort of TMAs was established in the lab with ccRCC that were collected immediately after nephrectomy, deidentified by the tissue procurement core at RPCI and received by the lab. The tissues were fixed in formalin, placed in a multi-tissue cassette, paraffin embedded and used for HDAC 1 and HDAC6/ERα staining. A larger cohort of patients obtained from the pathology core facility containing 120 ccRCC (GuCa2) and 88 metastatic ccRCC (GuCa4), were additionally analyzed for HDAC 1 expression. For TMAs and paraffin embedded formalin fixed tissue, the slides were first deparaffinized in xylene and decreasing concentrations of ethanol; antigen retrieval was carried out by boiling the slides in 10 mM sodium citrate in a microwave. The slides were washed, incubated with 3 % hydrogen peroxidase to inhibit peroxidase activity, blocked with horse serum (Vector Laboratories, Cat. no. S1000) and incubated with HDAC 1 or HDAC 6/ERα primary antibody overnight. For immunohistochemistry, slides were washed the next day, incubated with secondary HRP-conjugated antibody (Vector Laboratories, Cat. no. MP7401) followed by incubation with DAB (Dako, Cat. no. 3467), hematoxylin counterstaining, dehydration and mounting on coverslips using cytoseal. For immunofluorescence, slides were washed the next day, incubated with secondary antibodies conjugated to fluorochromes, followed by incubation with DAPI for nuclear staining and mounted on coverslips using Vectashield. Brightfield and fluorescent images were taken on Zeiss microscope with Axiovision software. HIF-1α and HIF-2α status in the TMA was obtained from Chintala *et al.* [19] and correlated with HDAC 1 status in these tumors.

### Statistical analyses

The outcomes measures (HDAC expression, cell counts, acetylated α-tubulin levels, and qPCR data) of the *in vitro* studies are reported by experimental group using the mean and standard deviation; and graphically using dot- or mean-plots. Comparisons are made between experimental groups using the paired *T*-test (when comparing expression between tumor and adjacent non-tumor tissue) and the two-sample Student’s *T*-test or one-way ANOVA, as appropriate. In the TMA samples, the association between the HDAC expression and HIF-1α/HIF-2α status was assessed using one-way ANOVA; and reported graphically using box-plots. The association between HDAC expression and tumor stage (T1-2 versus T3-4) or grade (I/II versus III/IV) was assessed using the Student’s *T*-test. The survival outcomes (overall, disease-specific, and progression-free survival) were summarized by HDAC mRNA status (unaltered versus upregulated) using standard Kaplan-Meier methods, with comparisons made using the log-rank test. Analyses were completed in SAS v9.4 (Cary, NC) at a significance level of 0.05; therefore a p-value less than 0.05 is considered statistically significant.

### TCGA data analysis

cbioportal website was used to analyze the TCGA ccRCC provisional database [20, 21]. ccRCC tumors with HDAC 1 and HDAC 6 overexpression were selected for overall and progression free survival.

### Ethics statement

All patients gave written informed consent for the collection of biomaterials. The study was approved by the Genitourinary Disease Site Research Group at Roswell Park Cancer Institute (number: BDR 036713).

## Results

### Class I and II HDACs are overexpressed in a subset of ccRCC tumors

**Fig. 1:**
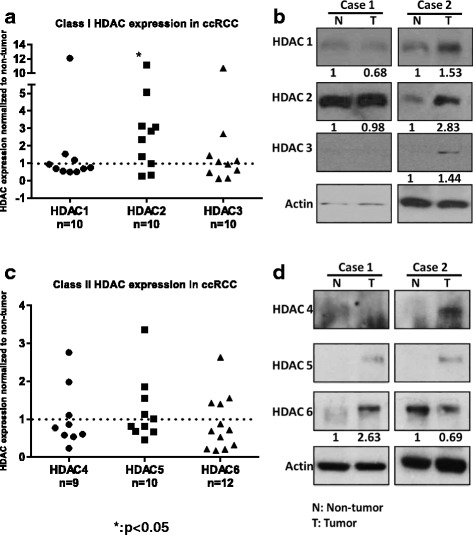
Class I and II HDACs are overexpressed in a subset of ccRCC tumors. Tissues from ccRCC tumors and adjacent non tumor tissues were homogenized and 50 μg of total protein was analyzed by Western blot for the expression of class I and II HDACs. **a**) Semi-quantitative analysis of HDAC bands were performed in Image J. Each tumor was normalized to GAPDH before normalizing it to their respective non-tumor tissue. **b**) Representative Western blots of two different matched tumor and non-tumor tissues show differential expression of class I HDACs. **c**) Semi-quantitative analysis of HDAC bands were performed in Image J. Each tumor was normalized to GAPDH before normalizing it to their respective non-tumor tissue. **d**) Representative Western blots of two different matched tumor and non-tumor tissues show differential expression of class II HDACs. The dotted line at 1 indicates the expression of HDAC 1 in adjacent non-tumor tissue. *p < 0.05 indicates statistically different HDAC 2 protein expression in tumor tissues as compared to the non-tumor tissue

To assess the expression of class I and II HDACs, we examined a set of ccRCC tumors and compared them to adjacent non-tumor tissue. Western blots were semi-quantified through Image J analysis, and HDAC band density in tumor tissue was normalized to its expression in the adjacent tissue for comparative analysis. As previously reported [6], class I HDAC expression was generally upregulated in ccRCC when compared to the non-tumor tissue (Fig. 1a and b). Interestingly, class II HDACs were downregulated in most of the tumor samples, but HDAC 6 was overexpressed in a small subset of ccRCC patients (Fig. 1c and d).

### HDAC 1 and HDAC 6 increase invasion and motility of renal tumor cell lines *in vitro*

**Fig. 2:**
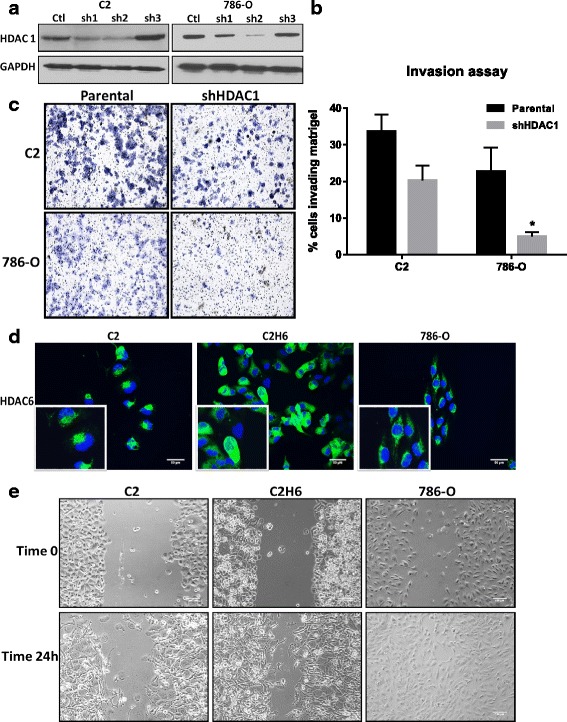
HDAC 1 and HDAC 6 increase cell invasion and migration in RCC cells, respectively. HDAC 1 was knocked down in 786–0 and C2 cells using retroviral supernatants. **a**) Three different clones with HDAC 1 knock down were generated and Sh2 was chosen in both cell lines for further experiments. **b**) BD biocoat matrigel chambers were used for measuring the invasion capacity of parental and HDAC 1 knocked down cells. C2 cells were incubated in the chamber for 24 h, whereas 786–0 cells were incubated for a short time point of 6 h. Cells at the bottom of the wells were visualized by crystal violet staining. **c**) Cells at the bottom of the well were counted blindly using a bright field microscope. *p < 0.05 indicates statistically different number of cells in shHDAC1 cells as compared to the parental cells. The error bars represent standard errors from biological triplicate experiments with technical replicates within each experiment. **d**) Representative images of renal tumor cell lines analyzed for HDAC 6 expression by immunofluorescence are shown. Scale bar indicates 50 μM distance and images are taken at 20X magnification. **e**) Representative images of scratch assays performed on C2, C2 overexpressing HDAC 6 and 786-O cells at time 0 and at the end of 24 h are shown

To assess the biological role of HDAC 1, we knocked down its gene expression in VHL-null renal tumor cell lines, and selected the clones that displayed the most efficient HDAC 1 protein knockdown (Fig. 2a). Surprisingly, the inhibition of HDAC 1 gene expression did not have significant effects on cell proliferation (data not shown). To analyze whether HDAC 1 knockdown affected the invasive capacity of the cells, we utilized BD biocoat matrigel chambers and counted the tumor cells that migrated through the membrane. The studies conducted with 786–0 and C2 renal cancer cell lines showed that knockdown of HDAC 1 led to reduced invasive capacity (Fig. 2b and c). The effect of HDAC 6 on cell motility was measured by scratch assays. C2 cells overexpressing HDAC 6 (C2H6) had increased motility at 24 h after scratching, as compared to the parental cell lines (Fig. 2d and e). 786–0 cells had higher motility than the other cell lines, as indicated by the faster scratch closure at 24 h (Fig. 2e).

### HDAC 1 and HDAC 6 increase invasiveness and motility through increased MMP2/9 activity and decreased acetylated α-tubulin, respectively

**Fig. 3:**
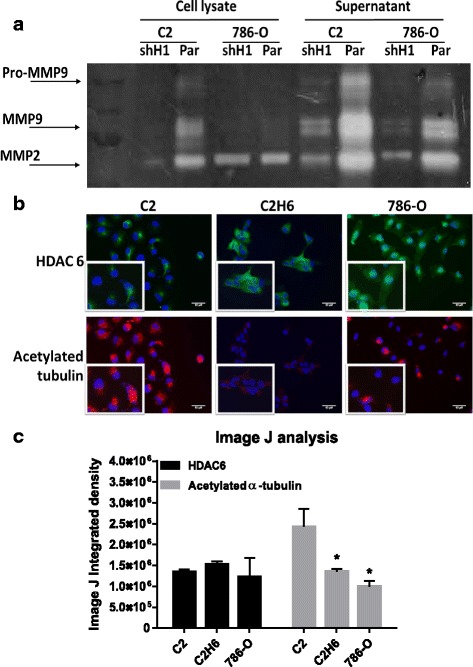
HDAC 1 and HDAC 6 increase MMP-2/9 activity and decrease acetylated α-tubulin levels, respectively. **a**) C2 and 786–0 parental and knocked down HDAC 1 were analyzed for MMP activity by gelatin zymography assay. MMP activity was measured both in cell lysates as well as cell supernatants. **b**) Renal tumor cell lines were analyzed for HDAC 6 and acetylated α-tubulin expression by immunofluorescence. The staining in red indicates acetylated α-tubulin and the staining in green indicates HDAC 6 expression. Scale bar indicates 50 μM distance and images are taken at 20X magnification. **c**) Image J analysis measured immunofluorescence by calculating integrated density values of at least three representative fields per cell line. *p < 0.05 indicates statistically significant difference in acetylated α-tubulin levels as compared to C2 cells. The error bars represent standard errors from triplicate experiments and p-value was calculated using students *t*-test

Class I HDACs increase invasive through increased MMP activity in different cancer cell lines [22–24]. Therefore, we analyzed the metalloproteinase and α-tubulin acetylation activity under our experimental conditions. Gelatin zymography assays revealed that HDAC 1-knockdown cells had lower MMP activity in both the supernatant and cell lysates (Fig. 3a). Interestingly, the Broad-Novartis cancer cell line encyclopedia of gene expression analysis of renal tumor cell lines shows that 786–0 cells indeed have higher HDAC 1 gene expression as compared to the C2 cells, with a corresponding increase in MMP-2/9 gene expression (Additional file 1: Figure S1). We also measured acetylated α-tubulin in HDAC 6-overexpressing and parental cells using an immunofluorescence assay. HDAC 6-overexpressing cells (C2H6) showed lower acetylated α-tubulin intensity as compared to parental C2 cells (Fig. 3b and c). In addition, 786–0 cells showed high HDAC 6 activity, as evidenced by lower acetylated α-tubulin levels (Fig. 3b and c).

### HIF-α regulates HDAC 1 expression in renal cell lines *in vitro*

**Fig. 4:**
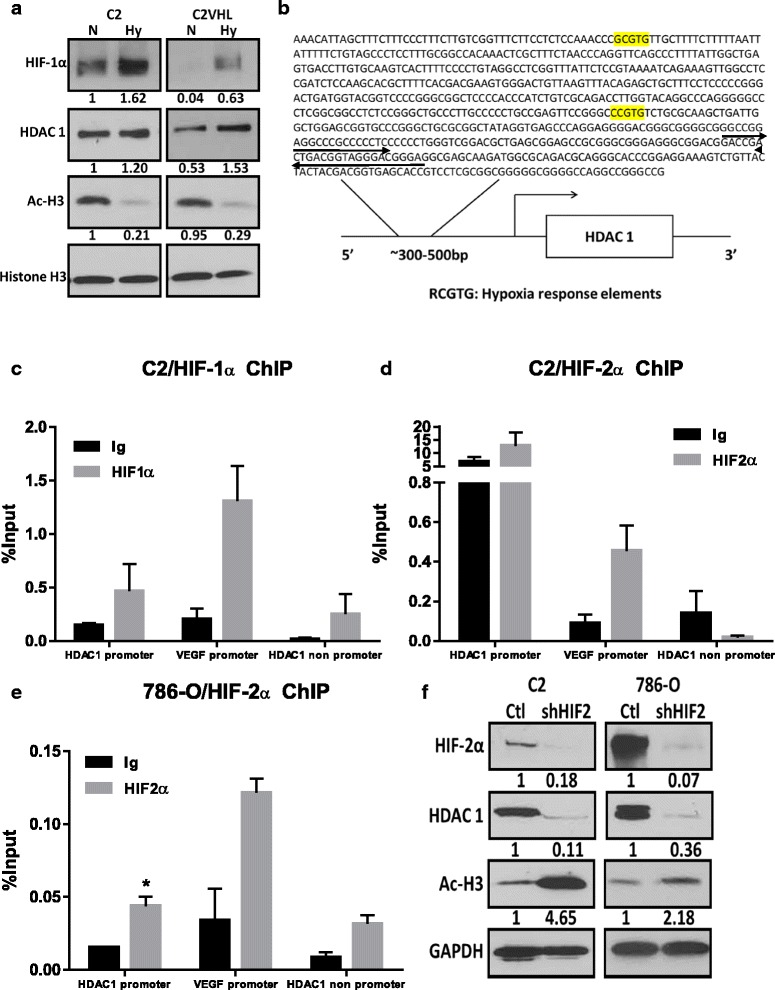
HDAC 1 expression is regulated by HIF in RCC cell lines. **a**) HDAC 1 expression was compared between C2 and C2VHL cells under normoxic and hypoxic conditions (mimicked by the use of 100 μM cobalt chloride) after overnight serum starvation. The numbers below the bands represent densitometry performed by Image J analysis on representative immunoblots relative to C2 bands in normoxic conditions with total histone H3 serving as loading control. **b**) HDAC 1 promoter region was analyzed for the presence of hypoxia response elements (HREs) by using the genomatix software. HREs represented by RCGTG and are highlighted in yellow and the black arrow represent the location of the forward and reverse primer. **c**-**e**) Chromatin immunoprecipitation assays were carried out in VHL null cell line C2 that expressed both HIF isoforms and 786–0 that only expressed HIF-2α. Pull downs with HIF isoforms were analyzed for HDAC 1 promoter/non-promoter region (1 kb upstream of the transcription start site) and the VEGF promoter (a known target of HIF) by qPCR. The error bars represent standard errors from biological duplicate experiments with technical replicates within each experiment. **f**) HIF-2α was knocked down in both models of renal cell line tumors by mammalian shRNA and analyzed for HDAC 1 expression and acetylated histone H3 by Western blot. The numbers below the bands represent densitometry performed by Image j analysis on representative immunoblots relative to parental cell lines in normoxic conditions with total GAPDH serving as loading control

To determine whether the VHL-HIF axis regulates HDAC 1 expression, we generated two isogeneic RCC cell lines with restored VHL, C2VHL and 786-0VHL expression. Western blot analysis revealed that HDAC 1 was differentially expressed in renal tumor cell lines with wild type and non-functional VHL *in vitro* (Additional file 1: Figure S2a). VHL-null cells showed higher HDAC 1 expression levels, as compared to their wild type VHL counterparts. This increase in HDAC 1 expression was also associated with greater HIF expression in VHL-null cell lines (Additional file 1: Figure S2a). The reintroduction of VHL in both cell lines, however, did not significantly alter the proliferation rate (data not shown). C2 cells displayed constitutive expression of HIF-1α and minimal expression of HIF-2α at 24 h (Additional file 1: Figure S2a). This constitutive expression of HIF-α was associated with higher HDAC 1 expression in C2 cells (Additional file 1: Figure S2a). In addition, upregulation of HDAC 1 protein expression in C2VHL cells by hypoxic stimulation was associated with an increase in HDAC 1 activity (measured by the levels of acetylated histone H3) (Fig. 4a and Additional file 1: Figure S6a-c). Similarly, HDAC 1 protein expression was induced by hypoxic mimetic agent cobalt chloride treatment in 786-0VHL cells (Additional file 1: Figure S2b). For quantitative analysis of protein expression, HDAC 1 protein levels in the different cell lines were measured by flow cytometry under both normoxic and hypoxic conditions. The HDAC 1 levels were measured as percentage of cells that were positive for HDAC 1 (tagged with FITC), as observed in the flow cytometry data. A representative image of the flow cytometry data illustrates an induction of HDAC 1 expression in the wt-VHL cells upon hypoxic stimulation (Additional file 1: Figure S2c and d). In accordance with our observation, the genomatix software showed the presence of hypoxia response elements (HREs), as represented by RCGTG, in the HDAC 1 promoter region upstream of the transcription start site (Fig. 4b). Interaction between HDAC 1 and HIF isoforms were evaluated by chromatin immunoprecipitation (ChIP) assays in cell lines with constitutive HIF expression. In C2 cells, both HIF-1α and HIF-2α pull downs were enriched for the HDAC 1 promoter region, as analyzed by qPCR (Fig. 4c and d). The HIF-2α only cell line, 786–0, also demonstrated enrichment for the HDAC 1 promoter region upon HIF-2α pull down (Fig. 4e). VEGF is a known target of both HIF isoforms, and VEGF promoter primers were used as positive control for ChIP assays to detect effective pull downs for HIF (Fig. 4c-e). In order to explore the possibility of HIF-2α playing a dominant role in regulating the expression of HDAC 1, HIF-2α was knocked down in both C2 and 786–0 cells. Silencing of HIF-2α in both cell lines dramatically reduced the protein levels of HDAC 1 that corresponded to increased acetylated histone H3, indicating loss of HDAC 1 activity in these cells (Fig. 4f and Additional file 1: Figure S6d-f).

### ER-α regulates acetylated α-tubulin levels in renal tumor cells *in vitro* through its interaction with HDAC 6

**Fig. 5:**
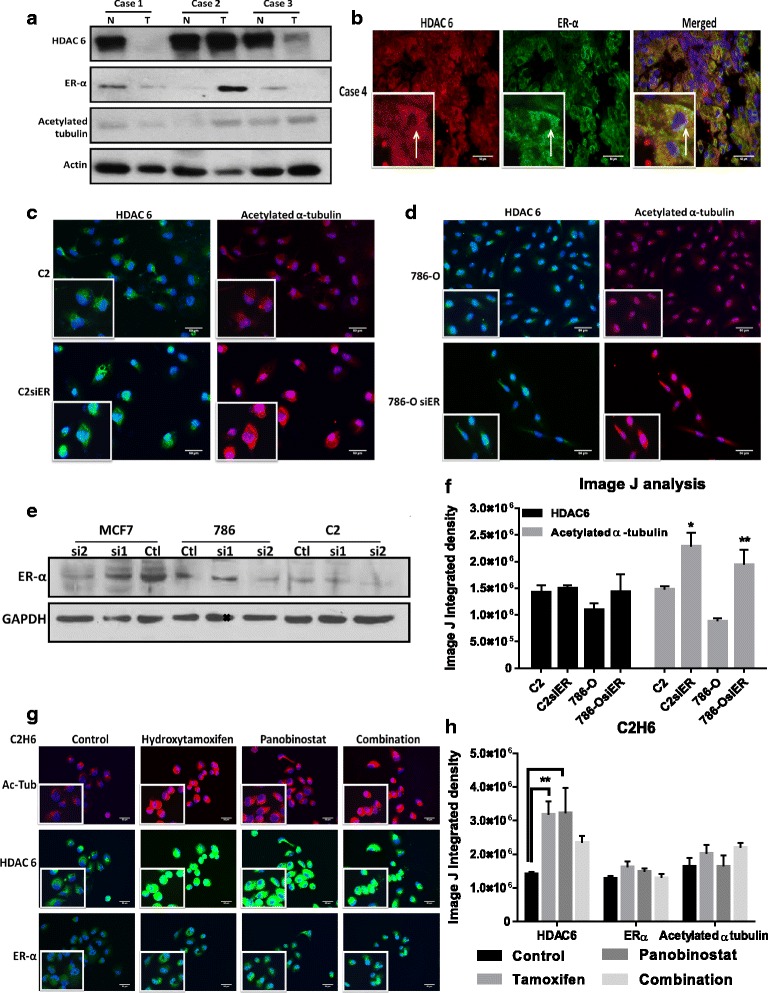
Acetylated α-tubulin is regulated by HDAC6/ER-α interaction in RCC cell lines. **a**) HDAC 6, ER-α and acetylated α-tubulin protein expression were measured by western blot analysis in ccRCC tumors and adjacent non-tumor tissue. **b**) A representative ccRCC tumor showed HDAC 6 (in red) and ER-α (in green) localization in the cytoplasm. **c**-**d**) Representative immunofluorescent images of acetylated α-tubulin (in red) and HDAC 6 (in green) expression in parental and ER-α knockdown cell lines are shown. **e**) Knockdown of ER-α in MCF-7 and renal cell tumors as measured by Western blot analysis. **f**) Image J analysis measured immunofluorescence by calculating integrated density values of at least three representative fields per cell line. *p < 0.05 and **p < 0.01 indicates statistically significant difference of acetylated α-tubulin levels in ER-α knockdown cells as compared to the parental cell lines. The error bars represent standard errors from triplicate experiments and p-value was calculated using students *t*-test. **g**) C2H6 cells treatment with 10 μM hydroxy tamoxifen and/or 50nM panobinostat for 4 h. Representative immunofluorescence images of acetylated α-tubulin (in red), HDAC 6 (in green) and ER-α (in green) are shown. **h**) Image J analysis measured immunofluorescence by calculating integrated density values of at least three representative fields per cell line. *p < 0.01 indicates statistically different HDAC 6 levels in treated cell lines as compared to control cells. The error bars represent standard errors from biological triplicate experiments with technical replicates for each experiment. Scale bar indicates 50 μM distance and images are taken at 20X magnification

HDAC 6 increases motility by deacetylation of α-tubulin and this increase is associated with ER-α expression in breast tumor cell lines [13]. Both HDAC 6 and ERα protein are expressed in ccRCC tumors, as measured by Western blot analysis (Fig. 5a). We further analyzed the subcellular location of HDAC 6 and ERα in ccRCC tumors and found that both HDAC 6 and ERα are expressed in the cytoplasm of these tumors in a punctuated format (Fig. 5b). We knocked down ERα expression in 786–0 and C2 cells (labeled as 786-OsiER and C2siER) to evaluate the role ERα in maintaining the deacetylated status of α-tubulin. The ER-positive breast cancer cell line MCF7 was used as a positive control for ERα. By using a siRNA pool mammalian vector, were able to successfully reduce ERα expression not only in the breast cancer cell line, but also the in the renal cell lines (Fig. 5e). ERα knockdown led to increased acetylated α-tubulin levels in both 786–0 and C2 cells, as observed by immunofluorescence (Fig. 5c-f). ERα silencing did not have an effect on HDAC 6 expression (as measured by integrated density of immunofluorescent images) (Fig. 5f). However, silencing ERα did not affect the proliferation rate of these cells (data not shown). Then, we treated C2, C2H6 and 786–0 cells with 10 μM hydroxytamoxifen and 50nM panobinostat (class I and class II HDAC inhibitor) for 4 h to analyze the effect of pharmacological inhibition of ERα and HDAC 6 on the levels of acetylated α-tubulin. We found that hydroxytamoxifen alone was able to increase acetylated α-tubulin, and the combination with panobinostat intensified this effect (Fig. 5g-h, Additional file 1: Figure S3a-d). In C2H6 but not in parental C2 cells, single and combination treatments increased HDAC 6 expression (Fig. 5h, Additional file 1: Figure S3b). Similarly, in 786-O cells single and combination treatments increased both HDAC 6 and ERα expression. However this increase in expression did not result in an increased HDAC 6 activity as measured by the presence of acetylated α-tubulin.

### HDAC 1 expression correlates with HIF expression in ccRCC and is associated with poorer survival

**Fig. 6:**
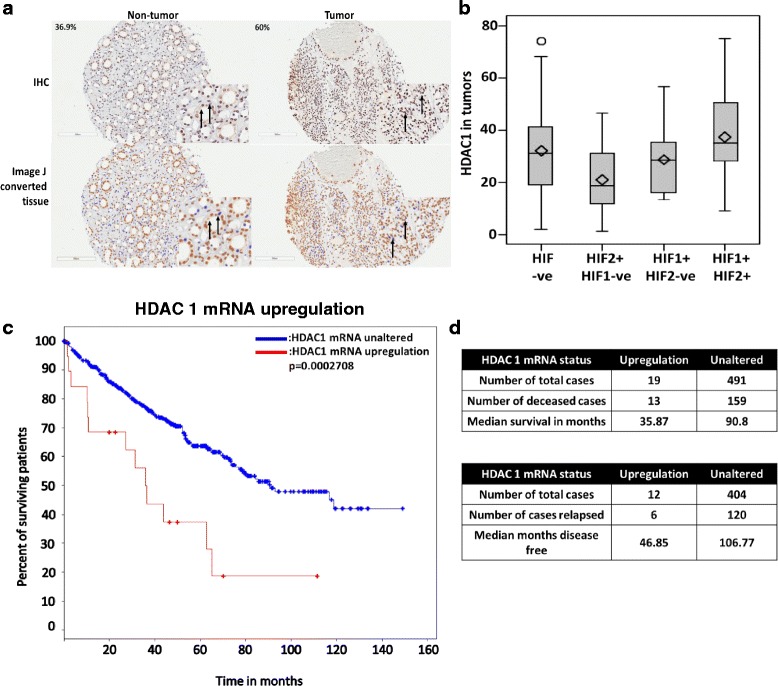
HDAC 1 and HIF positively correlate in clinical ccRCC. **a**) HDAC 1 expression in TMA was examined by immunohistochemistry. HDAC 1 positive nuclei were quantitated using Image J software and percent positive nuclei (indicated by brown staining) were used for further analysis. Black arrows indicate positive nuclei that were identified by the Image J software. The scale bar indicates 200 μM and the image was captured at 4X using the Aperio software. **b**) The association between HDAC1 and HIF1 & HIF2 statuses was examined using Wilcoxon rank sum and Kriskall-Wallis exact tests. All analyses were conducted in SAS v9.3 (Cary, NC) at a significance level of 0.05. **c**) Kaplan Meir curves (overall survival) of unaltered and upregulated *HDAC 1* mRNA levels in the TCGA data are shown. The blue line indicates unaltered *HDAC* 1 mRNA and the red line indicates upregulated *HDAC 1* mRNA levels. **d**) The table shows the numbers of patients in each group and survival data in months

We next interrogated a tissue microarray (TMA) consisting of matched tumor and adjacent non-tumor tissue for HDAC 1 expression and its correlation with HIF isoforms, as previously described [25] (Fig. 6a). Statistical analysis indicates that tumors that were HDAC 1-positive were more likely to be double positive for HIF-1α/HIF-2α (Fig. 6b). Surprisingly, however, HDAC 1 was not associated with tumor grade, overall survival or disease specific survival (data not shown). Furthermore, there was no association between survival status and either HIF isoforms in this cohort of tumors (data not shown). However, *HDAC 1* mRNA upregulation is present in 4 % of the patients in the TCGA data set [20, 21] and it is associated with worse overall survival (Fig. 6c-d). Both *HDAC 1* and *HDAC 6* mRNA upregulation are associated with higher tumor stage, though these differences are not statistically significant (Additional file 1: Figure S4a and b) [20, 21].

### HDAC 6 is associated with worse overall survival and progression free survival in TCGA data set

Although not statistically significant, *HDAC 6* mRNA upregulation (in 9 % of tumors) was associated with worse overall survival and progression free survival in ccRCC patients (Additional file 1: Figure S5a-d) [20, 21].

## Discussion

**Fig. 7:**
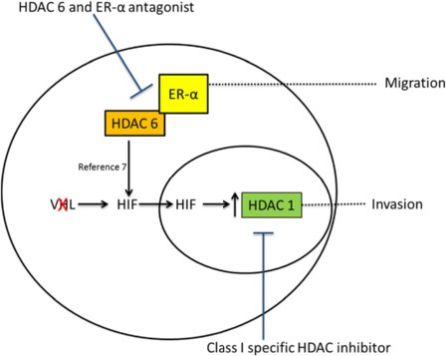
Schema of HDACs, hypoxia inducible factors and ER-α in clear cell renal cell carcinoma. Our studies show HDAC 1 can be upregulated by hypoxia inducible factors that is in turn stabilized by class II HDACs. Class II HDACs, specifically HDAC 6 can interact with ER-α. These interactions lead to increased motility and invasive capacity of ccRCC cell lines

In this study, we report that HDAC 1 and HDAC 6 modulate the motility and invasion of RCC cells *in vitro* through the regulation of matrix metalloproteases (MMPs) and acetylated α-tubulin, respectively (Fig. 7). In addition, we observed that HIF-α regulates HDAC 1 expression, while HDAC 6 and ERα increase α-tubulin deacetylation. In addition, HDAC 1 expression is associated with HIF overexpression in ccRCC tumors, and *HDAC 1* mRNA upregulation in TCGA data set is associated with poor outcome. *HDAC 6* mRNA upregulation is also associated with shorter overall and progression free survival, although not statistically significant. Taken together, these results suggest that HDACs may play a role in the aggressiveness of ccRCC and that a subset of these tumors may be targetable with HDAC inhibitors.

The *VHL* tumor suppressor gene is deregulated in approximately 70 % of the sporadic cases of ccRCC, and its major role is to decrease the stability of HIF isoforms under normal oxygen levels [3, 26–28]. The VHL-HIF axis deregulation has been implicated in the activation of several oncogenic pathways in ccRCC [29–31]. HIF isoforms are stabilized by histone deacetylases and can be antagonized by the use of HDAC inhibitors [7, 10, 32]. However, there have been only a few studies assessing the role of HIF isoforms in regulating HDAC expression and activity. A subset of ccRCC tumors overexpresses class I and II HDACs when compared to adjacent non-tumor tissues. Class I HDACs, HDAC 1 and HDAC 2, have been shown to increase invasion through the upregulation of MMP-2 and MMP-9 in different cancer cell lines [22, 24, 33]. TSA exhibits anti-invasive properties through upregulation of RECK, which further inhibits MMP-2 and MMP-9 in a variety of cell lines, including breast cancer cell lines and hepatocytes, amongst other cancer types [23, 24]. Our study demonstrates that HDAC 1 knockdown reduces the invasive capacity of renal tumor models through decreased MMP activity in these cells. On the other hand, HDAC 6 is known to be overexpressed in different tumor types, to have oncogenic functions and to interact with other pathways to enhance tumorigenesis [34–37]. 786–0 cells were associated with lower acetylated α-tubulin, suggesting increased HDAC 6 activity as compared to C2 cells. The clinical relevance of this is not yet fully understood; however, the 786–0 cells are thought to represent a more aggressive type of ccRCC as compared to C2 cells. Upon enhancing HDAC 6 activity in the C2 cells by using an overexpression system, we observed that HDAC 6 was associated with not only decreased acetylated α-tubulin levels but also with functional consequences in terms of enhanced cell motility. Thus, it can be speculated that those tumors with higher HDAC 1 or HDAC 6 expression have a potential to be more aggressive as well as more metastatic in the clinical setting.

Further investigation of the regulation of HDAC 1 revealed a possible role of HIF isoforms in ccRCC cell lines. When comparing VHL-null cell lines with wild type VHL cell lines of renal tumor models, HDAC 1 expression was found to be upregulated in VHL-null cell lines. In addition, hypoxia stimulation of wt-VHL cells led to both HIF and HDAC 1 upregulation. Chromatin immunoprecipitation assay findings showed that the HDAC 1 promoter region is enriched upon pull down of both HIF isoforms and knockdown of HIF-2α in renal tumor cell lines reduced HDAC 1 expression in these cells. HDAC 1 activity as measured by acetylated histone H3 is also affected in these cell lines. Therefore, the above results indicate that HIF is involved in the regulation of HDAC 1 protein expression and activity. To date, anti-angiogenic drugs and agents inhibiting the mTOR pathway have been extensively studied in ccRCC treatment. Targeting HDACs in ccRCC, therefore, has the potential to reduce not only HDAC activity but also reduce HIF stability. This may further decrease HDAC expression and activity by HIFs.

The examination of ccRCC tumor samples obtained by nephrectomies demonstrated overexpression of HDAC 6 in a subset of tumors along with ERα expression. This phenomenon occurs regardless of the gender of the patient, indicating that ERα may have a potential role in the biology of ccRCC tumors. By immunofluorescent microscopy analysis of the expression of these proteins in the tumor, both ERα and HDAC 6 were present exclusively in the cytoplasm. In breast cancer, the colocalization of HDAC 6 and ERα in the cytoplasm has been associated with better clinical outcome in tamoxifen-treated patients as well as increased deacetylation of α-tubulin, which led to enhanced cell motility *in vitro* [13, 14]. Thus, we carried out ERα knockdown assays to examine the putative role of ERα in renal tumor cell lines, in particular the role on deacetylation of α-tubulin. Knockdown of ERα in renal tumor cell lines drastically increased the levels of acetylated α-tubulin, confirming that both HDAC 6 and ERα regulate the levels of acetylated α-tubulin and, thus, play roles in regulating the motility of these cells. Thus, the HDAC 6/ER-α interaction represents a potential therapeutic target, for reducing the metastatic potential of ccRCC. Pharmacological inhibition of HDAC 6 and ERα in renal tumor cell lines showed similar effects as ERα knockdown in these cells. Tamoxifen showed enhanced effects on α-tubulin acetylation in 786–0 cells, and this effect was greater with the addition of panobinostat. Single agent and combination treatments increased HDAC 6 and ER-α expression in C2H6 and 786-O cells; however this did not result in increased HDAC 6 activity. The mechanisms of increased ERα and 786-O expression by treatments are not entirely known. These results indicate that in ccRCC, tamoxifen treatment may reduce metastatic potential. Furthermore, when combined with an HDAC inhibitor, such as panobinostat (that has cytotoxic effects), can lead to both anti-tumor effects and reduced metastasis. Moreover, in 100 ccRCC tumors, HIF-1/2α and HDAC 1 positively correlated with one another; however, HDAC 1 expression did not correlate with overall or disease free survival. TCGA data further revealed that HDAC 1 overexpression is associated with worse overall survival and higher tumor stage (not statistically significant). Hence, targeting HDAC 1 by using class I specific HDAC inhibitors may not only reduce the invasiveness of the disease, but the level of HDAC 1 itself can be used as a prognostic indicator in ccRCC. Similarly, *HDAC 6* mRNA upregulation, although not statistically significant, showed a trend towards worse overall survival as well as higher tumor stage in ccRCC patients. Therefore, targeting HDAC 6 using a class II specific HDAC inhibitor may reduce the motility and aggressiveness of ccRCC tumors.

Although HDAC inhibitors have shown potential anti-tumor activities in preclinical models, the clinical development of this class of drugs has only achieved moderate success in hematological malignancies and not in solid tumors. However, these agents have been tested primarily as single agents in solid tumors and combination with already-approved therapies have not been extensively studied. In ccRCC, our lab has previously shown that the class I HDAC inhibitor, Entinostat, enhances immunotherapy [38] and another study demonstrated synergistic anti-proliferative effects of the combination of sorafenib with HDAC inhibitors [39]. The subset of ccRCCs that express high levels of HDAC 1 and HDAC 6 may be the most suitable patient population for rational combination strategies using HDAC inhibitors with other agents.

## Conclusions

Our study indicates a potential use for HDAC 1 and HDAC 6 expression status in identifying a subset of ccRCC patients who are suitable for treatment with HDAC inhibitors. Clinical testing of histone remodeling drugs in rational combination strategies will shed additional light on the potential therapeutic value of this class of agents in ccRCC.

## Additional file

Additional file 1:
**Figure S1**. VHL, HIF, HDAC1 and related gene expression in renal tumor cell lines. Gene expression analysis of renal tumor cell lines using the Broad-Novartis cancer cell line encyclopedia show the levels of players in the VHL-HIF axis. The top row indicates the gene analyzed in different renal tumor cell lines (in the left most column). Red color indicates gene upregulation and blue color indicates downregulation of genes in the renal tumor cell lines. **Figure S2**. Hypoxia induces HDAC 1 expression in clear cell renal tumor cell line. a) Parental VHL null cells C2 and 786–0 were compared to cells with wt-VHL introduced for HIF-1α, HIF-2α and HDAC 1 protein expression. The left panel measures protein expression in C2 isogeneic cell lines and the right panel measures protein expression in 786–0 isogenic cell lines. The numbers below the bands represent densitometry performed by Image J analysis on representative immunoblots relative to their respective isogeneic VHL null cells with GAPDH serving as a loading control. b) HDAC 1 expression was compared between 786–0 and 786-0VHL cells under normoxic and hypoxic conditions (mimicked by the use of 100 μM cobalt chloride) after overnight serum starvation. The numbers below the bands represent densitometry performed by Image J analysis on representative immunoblots relative to 786–0 bands in normoxic conditions with total GAPDH serving as loading control. c-d) HDAC 1 protein expression was quantitatively measured by flow cytometry under normoxic and hypoxic conditions. **p* < 0.05 indicates statistically different HDAC 1 expression in wt-VHL cells as compared to VHL null cell lines. The error bars represent standard errors from biological triplicate experiments with technical replicates within each experiment. e) The figure is a representative image obtained from flow cytometry. The Y-axis represents HDAC 1 expression measured by FITC and Y-axis represents cell cycle measured by propidium idodide. **Figure S3**. Panobinostat and tamoxifen treatment increases acetylated α-tubulin in renal tumor cell lines. a-d) C2 and 786–0 cells were treated with 10 μM hydroxy tamoxifen and/or 50nM panobinostat for 4 h. Representative immunofluorescence images of acetylated α-tubulin (in red), HDAC 6 (in green) and ER-α (in green) are shown. Image J analysis measured immunofluorescence by calculating integrated density values of at least three representative fields per cell line. **p* < 0.05 indicates statistically different acetylated α-tubulin levels in treated cells as compared to the control cells. The error bars represent standard errors from biological triplicate experiments with technical replicates for each experiment. **Figure S4**. HDAC 1 and HDAC 6 mRNA expression are associated with higher stage of ccRCC. a) *HDAC 1* and b) *HDAC 6* mRNA levels in the TCGA data set were measured by RNA-sequencing data. T1-T2 represent lower ccRCC stage whereas T3-T4 represent higher ccRCC stage where the tumor has invaded the adjacent organs or metastatic disease. P-values were calculated by using the students *t*-test. **Figure S5**. HDAC 6 expression and overall or progression free survival. a) Kaplan Meir curves (overall survival) of unaltered and upregulated *HDAC 6* mRNA levels in the TCGA data are shown. The blue line indicates unaltered *HDAC 6* mRNA and the red line indicates upregulated *HDAC 1* mRNA levels. b) Kaplan Meir curves (progression free survival) of unaltered and upregulated *HDAC 6* mRNA levels in the TCGA data are shown. c-d) The table shows the actual numbers of patients in each group and survival data/progression free survival data in months. e) *COL6A1* protein expression as measured by RRPA was compared between tumors with unaltered and upregulated *HDAC 6* mRNA levels. **Figure S6**. Image J quantitation of western blot shows inverse relation between hypoxia inducible factors and HDAC 1. a-c) Image J quantitation of western blots show induction of HIF-1α, HDAC 1 and reduction of acetylated histone H3 in hypoxic conditions as compared to normoxia in both C2 and 786-O isogenic cell lines. C2 Normoxia and 786-O Normoxia were used to normalize Image J quantitation. d-f) Image J quantitation of western blots show reduction in HDAC 1 and increase in acetylated tubulin upon HIF-2α knockdown in C2 and 786-O cells. C2 control and 786-O control cells were used to normalize Image J quantitation. Triplicate western blots were plotted with error bars indicating standard error of mean. **p* < 0.05 and ***p* < 0.01. (PPTX 2954 kb)
