# Supplementary material for: HDAC 1 and 6 modulate cell invasion and migration in clear cell renal cell carcinoma
Source: BMC Cancer. 2016 Aug 9;16:617. doi: 10.1186/s12885-016-2604-7 (PMC4977667; doi:10.1186/s12885-016-2604-7)
Supplement: Additional file 1: — Figure S1. VHL, HIF, HDAC1 and related gene expression in renal tumor cell lines. Gene expression analysis of renal tumor cell lines using the Broad-Novartis cancer cell line encyclopedia show the levels of players in the VHL-HIF axis. The top row indicates the gene analyzed in different renal tumor cell lines (in the left most column). Red color indicates gene upregulation and blue color indicates downregulation of genes in the renal tumor cell lines. Figure S2. Hypoxia induces HDAC 1 expression in clear cell renal tumor cell line. a) Parental VHL null cells C2 and 786–0 were compared to cells with wt-VHL introduced for HIF-1α, HIF-2α and HDAC 1 protein expression. The left panel measures protein expression in C2 isogeneic cell lines and the right panel measures protein expression in 786–0 isogenic cell lines. The numbers below the bands represent densitometry performed by Image J analysis on representative immunoblots relative to their respective isogeneic VHL null cells with GAPDH serving as a loading control. b) HDAC 1 expression was compared between 786–0 and 786-0VHL cells under normoxic and hypoxic conditions (mimicked by the use of 100 μM cobalt chloride) after overnight serum starvation. The numbers below the bands represent densitometry performed by Image J analysis on representative immunoblots relative to 786–0 bands in normoxic conditions with total GAPDH serving as loading control. c-d) HDAC 1 protein expression was quantitatively measured by flow cytometry under normoxic and hypoxic conditions. *p < 0.05 indicates statistically different HDAC 1 expression in wt-VHL cells as compared to VHL null cell lines. The error bars represent standard errors from biological triplicate experiments with technical replicates within each experiment. e) The figure is a representative image obtained from flow cytometry. The Y-axis represents HDAC 1 expression measured by FITC and Y-axis represents cell cycle measured by propidium idodide. Figure S3. Panobinostat and ta [file 12885_2016_2604_MOESM1_ESM.pptx]

## Slide 1
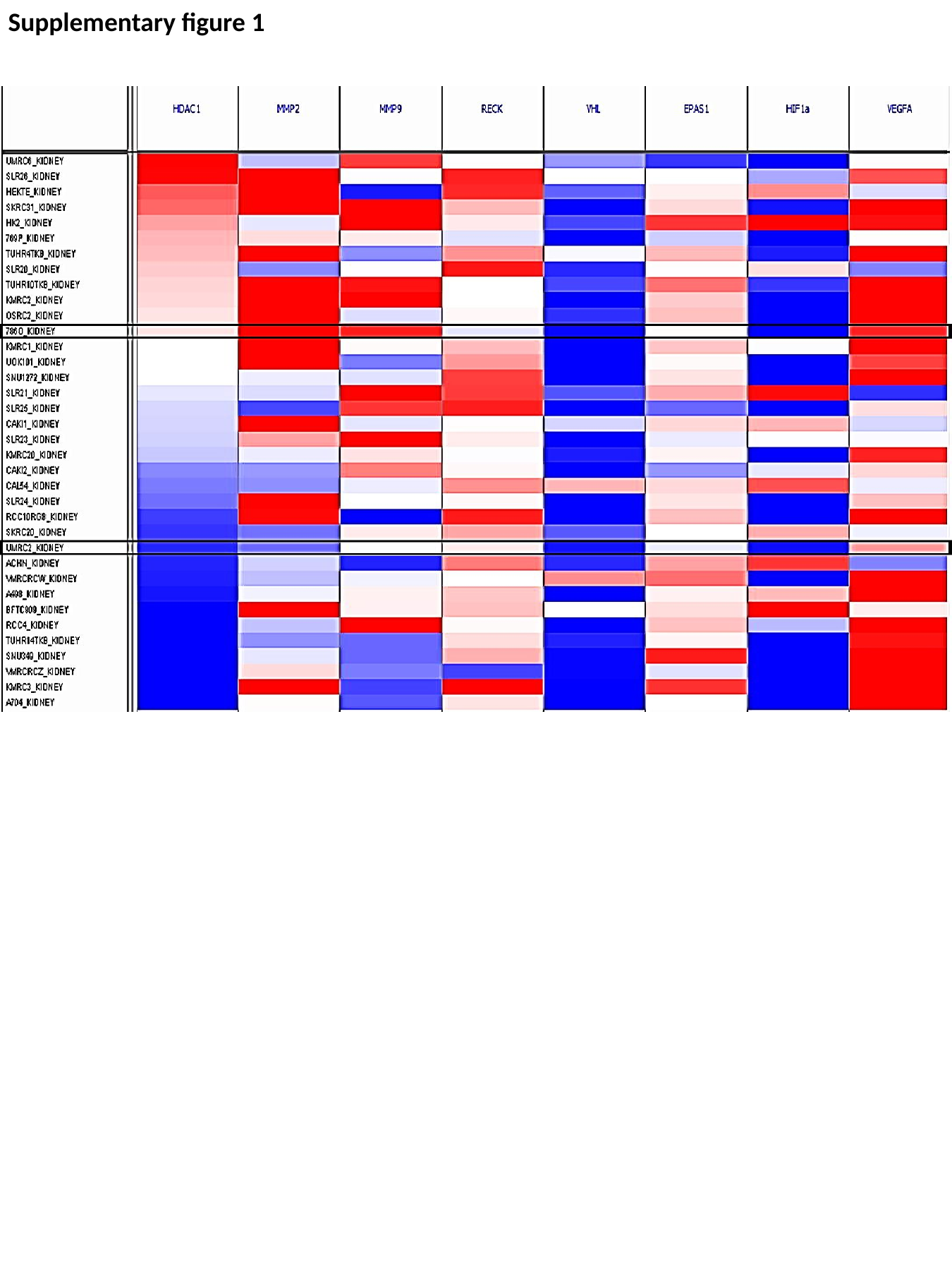

Supplementary figure 1

## Slide 2
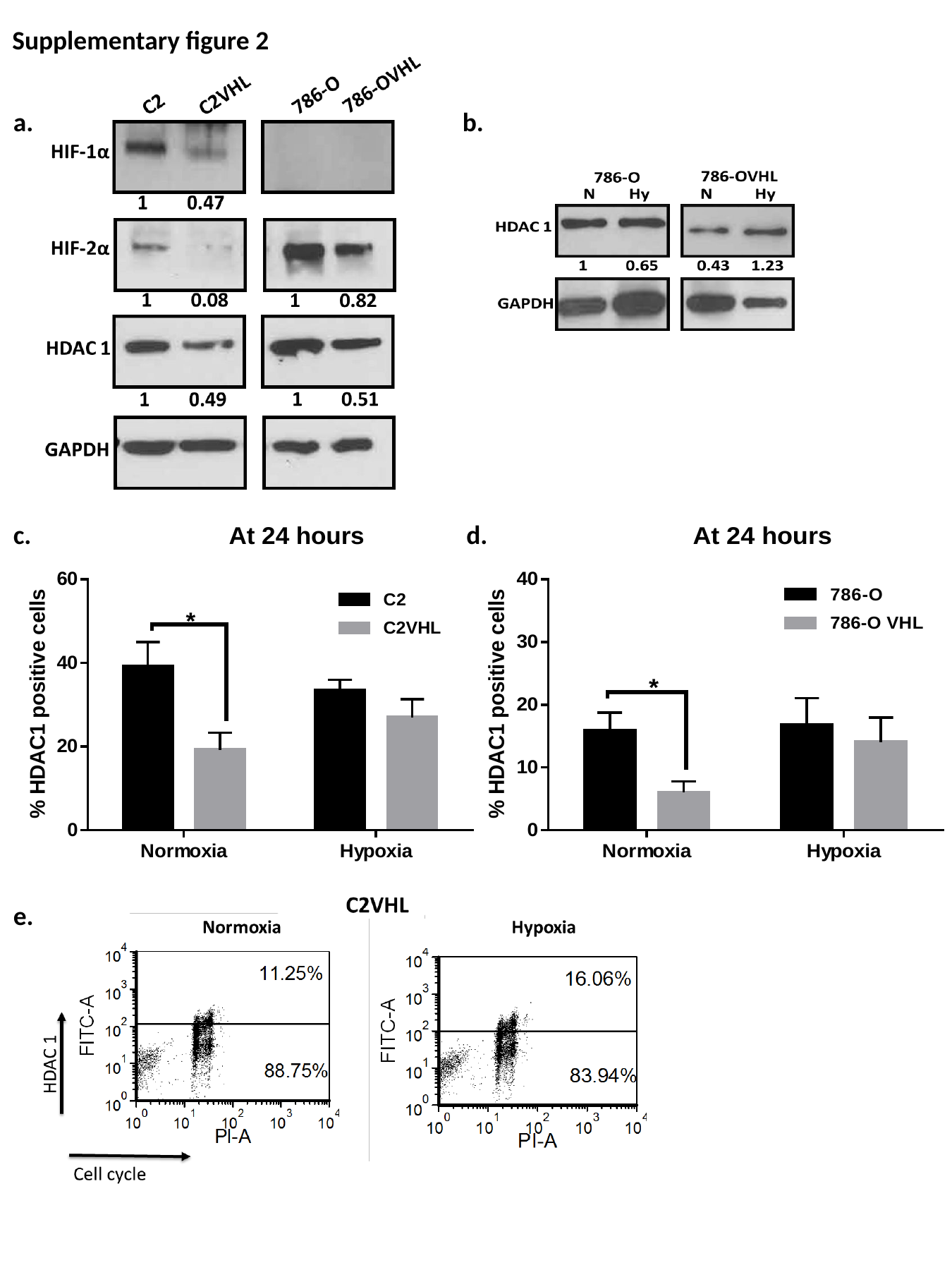

Supplementary figure 2
 b.
 d.
e.

## Slide 3
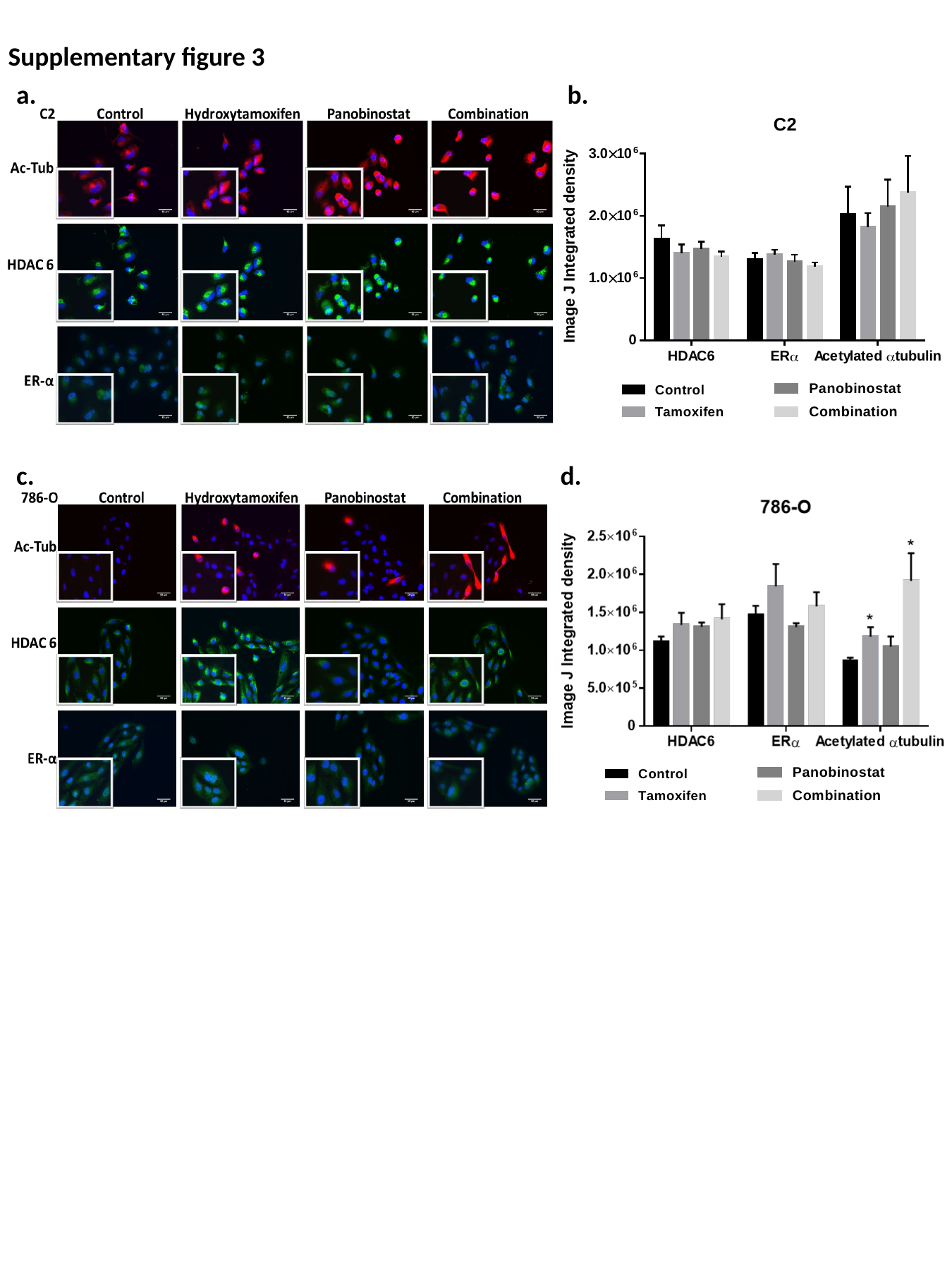

Supplementary figure 3
 b.
c. d.

## Slide 4
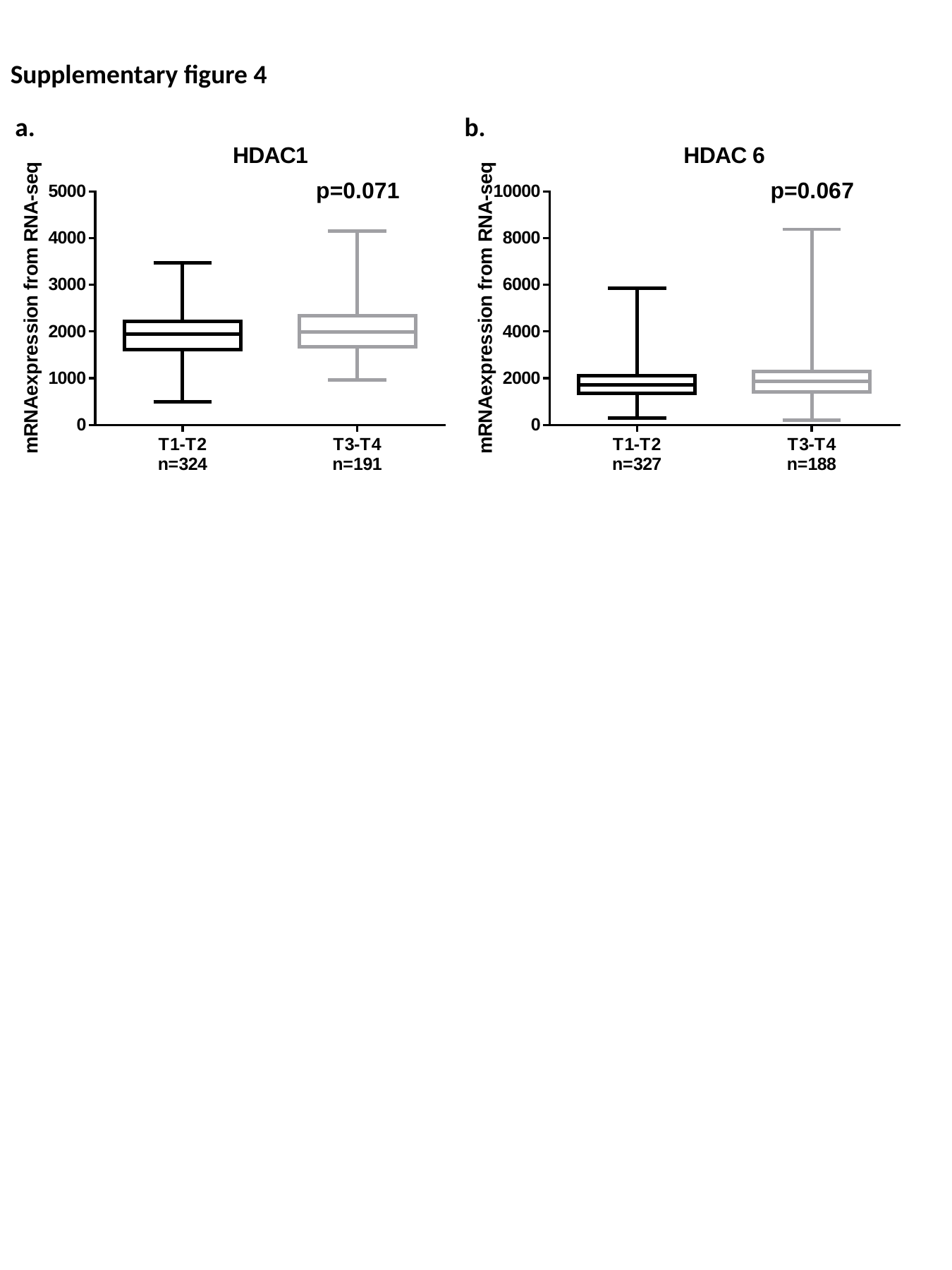

Supplementary figure 4
 b.

## Slide 5
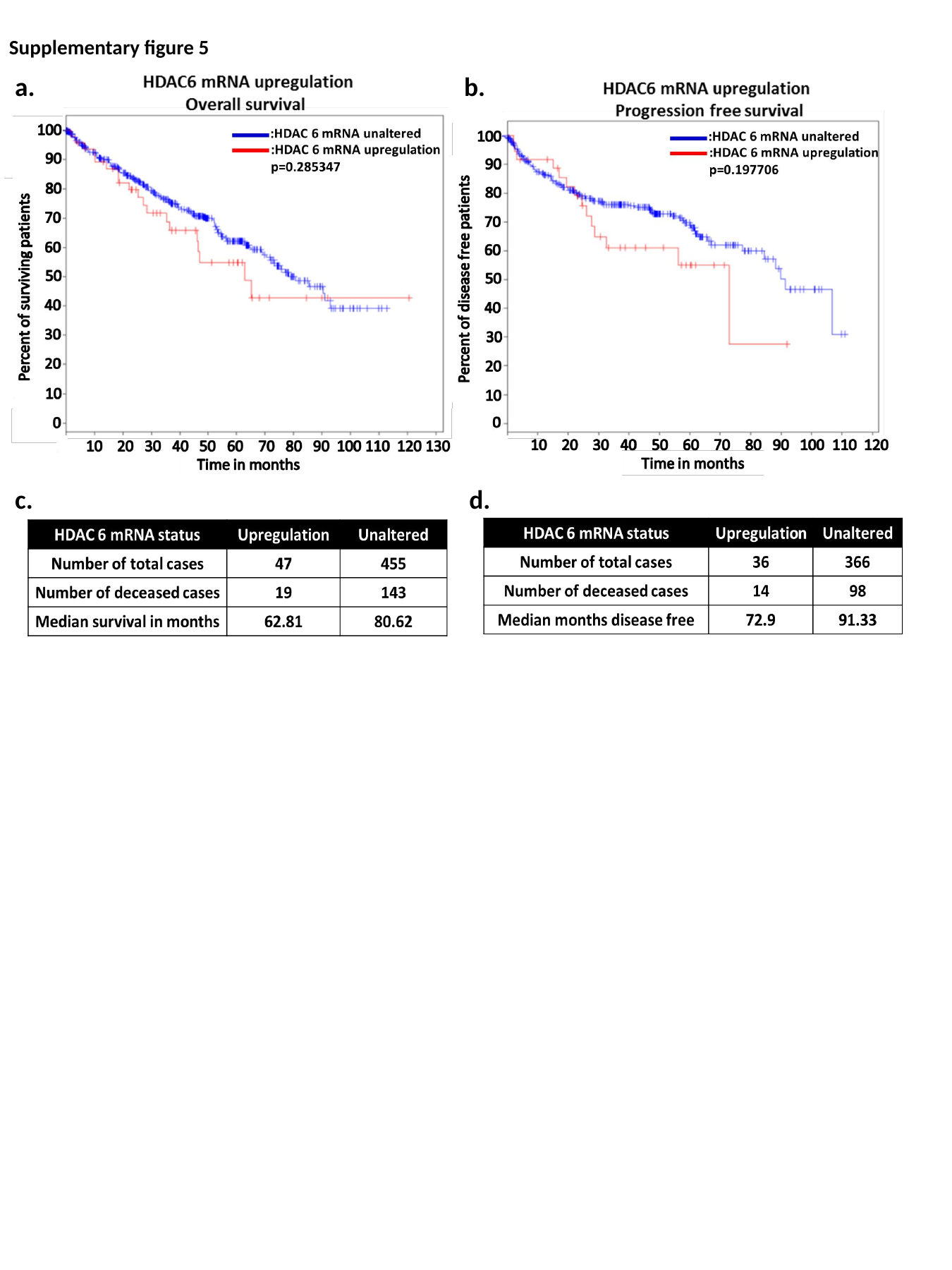

Supplementary figure 5
 b.
c. d.

## Slide 6
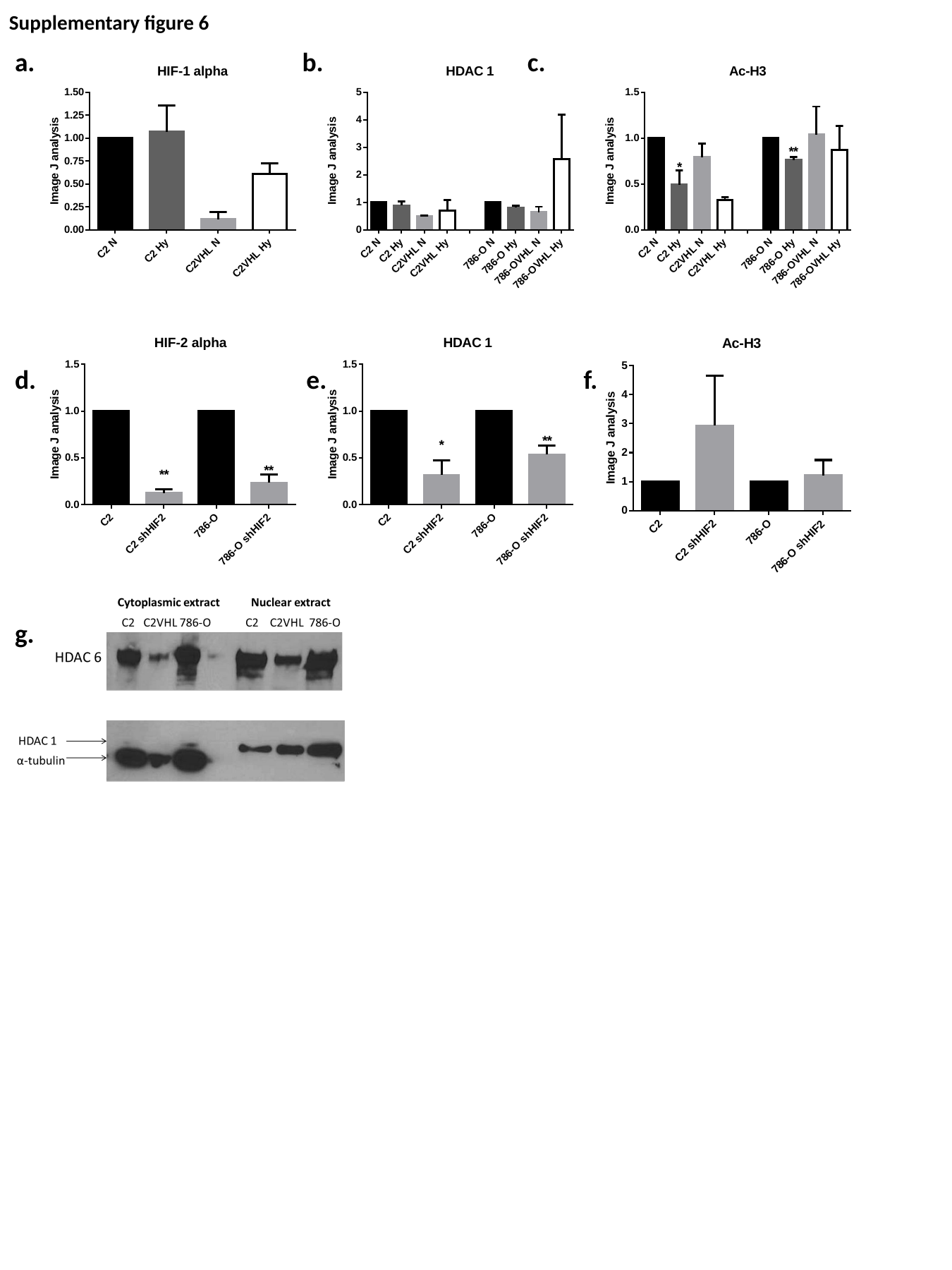

Supplementary figure 6
 b. 		 c.
 e. f.
g.
